# Microglial States Are Susceptible to Senescence and Cholesterol Dysregulation in Alzheimer's Disease

**DOI:** 10.1111/acel.70189

**Published:** 2025-08-11

**Authors:** Boyang Li, Shaowei Wang, Bilal Kerman, Cristelle Hugo, E. Keats Shwab, Chang Shu, Ornit Chiba‐Falek, Zoe Arvanitakis, Hussein N. Yassine

**Affiliations:** ^1^ Department of Neurology, Keck School of Medicine University of Southern California Los Angeles California USA; ^2^ Division of Translational Brain Sciences, Department of Neurology Duke University Medical Center Durham North Carolina USA; ^3^ Center for Genomic and Computational Biology Duke University Medical Center Durham North Carolina USA; ^4^ Center for Genetic Epidemiology, Department of Population and Public Health Sciences, Keck School of Medicine University of Southern California Los Angeles California USA; ^5^ Rush Alzheimer's Disease Center Rush University Medical Center Chicago Illinois USA; ^6^ Center for Personalized Brain Health University of Southern California Los Angeles California USA

**Keywords:** Alzheimer's, cellular senescence, cholesterol, microglia

## Abstract

Cellular senescence is a major contributor to aging‐related degenerative diseases, including Alzheimer's disease (AD), but much less is known about the key cell types and pathways driving senescence mechanisms in the brain. We hypothesized that dysregulated cholesterol metabolism is central to cellular senescence in AD. We analyzed single‐cell RNA‐seq data from the ROSMAP and SEA‐AD cohorts to uncover cell type‐specific senescence pathologies. In ROSMAP snRNA‐seq data (982,384 nuclei from postmortem prefrontal cortex), microglia emerged as central contributors to AD‐associated senescence phenotypes among non‐neuronal cells. Homeostatic, inflammatory, phagocytic, lipid‐processing, and neuronal‐surveillance microglial states were associated with AD‐related senescence in both ROSMAP (152,459 microglia nuclei from six brain regions) and SEA‐AD (82,486 microglia nuclei) via integrative analysis. We assessed top senescence‐associated bioprocesses and demonstrated that senescent microglia exhibit altered cholesterol‐related processes and dysregulated cholesterol metabolism. We identified three gene co‐expression modules representing cholesterol‐related senescence signatures in postmortem brains. To validate these findings, we applied these signatures to snRNA‐seq data from iPSC‐derived microglia（iMGs) exposed to myelin, Aβ, apoptotic neurons, and synaptosomes. Treatment with AD‐related substrates altered cholesterol‐associated senescence signatures in iMGs. This study provides the first human evidence that dysregulated cholesterol metabolism in microglia drives cellular senescence in AD. Targeting cholesterol pathways in senescent microglia is an attractive strategy to attenuate AD progression.

## Graphical abstract

Senescent microglia in Alzheimer's disease exhibit dysregulated cholesterol metabolism and express Disease‐Associated Microglia (DAM) signatures, including inflammatory and phagocytic markers. Single‐nucleus RNA‐seq from human brain tissues identified cholesterol‐related senescence gene co‐expression modules linked to microglial senescence. These signatures were validated in iPSC‐derived microglia exposed to AD‐relevant substrates, suggesting the transition of homeostatic and functional microglial states into senescent states with DAM markers, and highlighting cholesterol pathways as therapeutic targets to microglial pathology in AD.

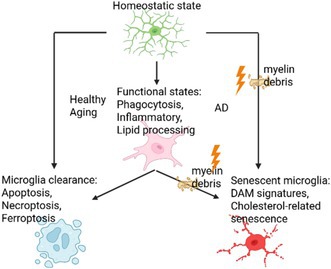

## 1 Introduction

Alzheimer's disease (AD) is a progressive neurodegenerative disorder, with aging recognized as the greatest risk factor (Hou et al. 2019). Aging is a complex process marked by the accumulation of cellular and molecular damage, driving tissue dysfunction and increasing susceptibility to diseases such as AD (López‐Otín et al. 2013). Among the hallmarks of aging is cellular senescence—a state of irreversible growth arrest often accompanied by a pro‐inflammatory secretory phenotype known as the senescence‐associated secretory phenotype (SASP) (Dodig et al. 2019). In AD, the brain accumulates senescent cells that contribute to neurodegeneration and cognitive decline by releasing inflammatory molecules and promoting inflammation.

Understanding how senescence contributes to AD pathology may open new avenues for intervention. Among the hallmarks of senescent cells are enlarged nuclei, lipid‐loaded lysosomes, and lipid droplets. Senescent cells exhibit deterioration in nuclear architecture, including the loss of nuclear lamina components such as lamin‐B1, and these morphological changes have been utilized in machine‐learning models to predict senescence with high accuracy (Heckenbach et al. 2022; Belhadj et al. 2023). Additionally, lipofuscin (SenTraGor, STG) staining has been used as a marker of senescent cells, labeling non‐degradable lipid and protein aggregates in the cytoplasm of aged or damaged cells (Salmonowicz and Passos 2017; Evangelou et al. 2017).

Oligodendrocytes—derived from oligodendrocyte progenitor cells (OPCs)—are specialized glial cells in the CNS responsible for producing myelin, a cholesterol‐enriched insulating sheath that wraps around the axon to facilitate neural transmission and provide metabolic support to axons (Kalafatakis and Karagogeos 2021) [Damage and Repair]. When myelin is degraded in AD, microglia, the brain's resident immune cells, recognize and engulf the myelin debris, promoting lipid‐droplet formation and adopting a pro‐inflammatory state—hallmarks of disease‐associated microglia (DAM) (Loving and Bruce 2020). The balance between clearance of myelin debris for remyelination and excessive inflammation plays an important role in maintaining brain cholesterol homeostasis (Gross et al. 2024). Senescent microglia are increasingly recognized as contributors to AD pathogenesis, and targeting microglial senescence may slow AD progression. In aging and AD mouse models, microglia can adopt a senescent phenotype marked by impaired phagocytosis, chronic inflammation, and altered gene expression. These cells display elevated markers such as p16^INK4a^ and p21^CIP1^, as well as a senescence‐associated secretory phenotype (SASP) enriched in pro‐inflammatory cytokines like IL‐6 and TNF‐α. Such senescent microglia accumulate around amyloid plaques and show reduced ability to clear pathological proteins, thereby exacerbating neurodegeneration. Notably, studies targeting removal of senescent glia can reduce tau pathology and improve cognitive outcomes, suggesting a causative role in disease progression (Hu et al. 2021; Bussian et al. 2018). While these findings offer important insights, most evidence for microglial senescence in AD comes from mouse models, and further work is needed to confirm their presence and functional impact in human disease.

Despite growing evidence linking senescence to AD (Herdy et al. 2022; Dehkordi et al. 2021; Fancy et al. 2024), many critical questions remain. It is unclear which brain cell types exhibit senescence in AD, how senescence contributes to disease pathology, and what factors mediate this process. Astrocytes, microglia, oligodendrocytes (OPCs), and vascular cells are all essential to brain health, yet their specific roles in AD‐related senescence remain poorly defined. Previously, we reported that oxidized cholesterol species 7‐hydroxycholesterol (7α‐OHC) are elevated in postmortem AD tissues and induce cellular senescence in astrocytes. Meanwhile, the cholesterol‐lowering drug cyclodextrin (CD) decreased senescence and neuroinflammation markers in APOE4‐TR mice (Wang et al. 2025). We hypothesize that excessive cholesterol‐related stressors in AD brains (myelin or 7α‐OHC) lead to microglial senescence, which contributes to inflammation and AD pathology. To address this hypothesis, we analyzed single‐cell RNA sequencing (scRNA‐seq) datasets from the well‐characterized ROSMAP and SEA‐AD cohorts, consisting of 427 (ROSMAP) and 84 (SEA‐AD) patients with and without AD. We further investigated the causal relationship between cholesterol dysregulation and cellular senescence in AD using iPSC‐derived microglia (iMGs).

## 2 Results

### 2.1 Heterogeneous Expression of Cellular Senescence Markers Across Glial Cell Types and Consensus Pathologic Measurement of AD

**FIGURE 1:**
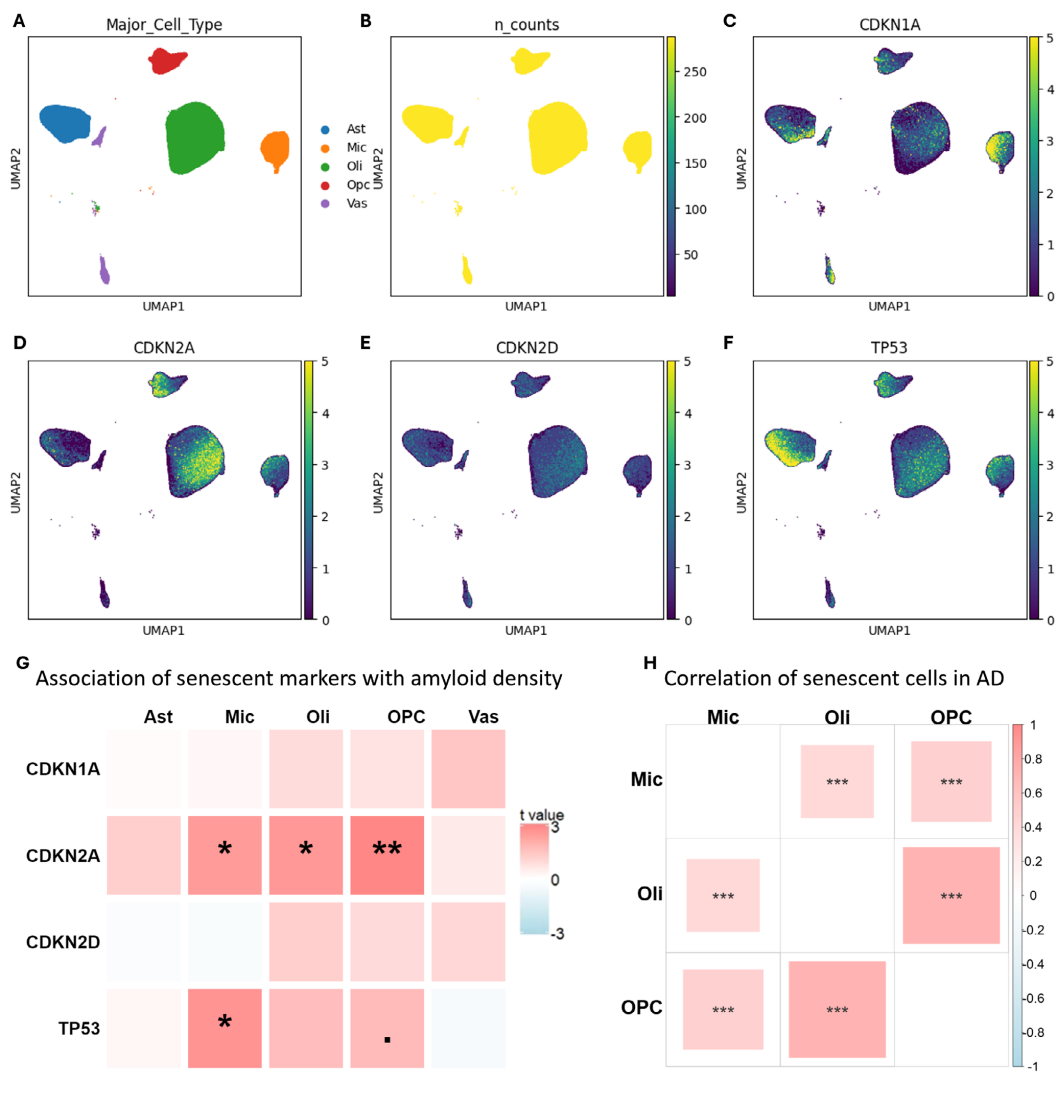
Different cell types have distinct senescence transcriptional signatures. (A–F) Normalized expression pattern of cellular senescence marker genes among non‐neuronal cell types (Ast: Astrocytes; Mic: Microglia; Oli: Oligodendrocytes; Opcs: Oligodendrocyte progenitor cells; Vas: Vasculature cells; n_counts: Equivalent total counts per cell after normalization). (G) Percentage expression (percentage of nuclei with the gene expression > 0) of cellular senescence marker genes among non‐neuronal cell types compared to amyloid density using zero‐inflated beta regression model. Data shown are color coded t value (the signed effect size divided by standard error) and “fdr” adjusted *p* value per cell type (.*p* < 0.1, *p < 0.05, **p < 0.01, ***p < 0.001). (H) Pearson‘s correlation of senescent (CDKN2A positive) cells. Data shown are color coded correlation coefficient and *p* value (.*p* < 0.1, *p < 0.05, **p < 0.01, ***p < 0.001).

The snRNA‐seq expression profile of 982,364 nuclei from the prefrontal cortex (PFC) of postmortem brain tissue from 427 participants (Table S1) in the ROSMAP (Religious Orders Study/Memory and Aging Project) was re‐analyzed, targeting cellular senescence‐related genes and pathways (Mathys et al. 2023). These cells include astrocytes (149,558), microglia (79,188), oligodendrocytes (645,142), oligodendrocyte precursor cells (OPCs; 90,502), and vasculature cells (17,974). Based on the postmortem AD diagnoses, which rely on the NIA‐AA consensus pathologic diagnosis for AD, the study included 239 AD and 188 non‐AD participants. To investigate cellular senescence across major glial cell types, we examined the expression patterns of several canonical cellular senescence markers—CDKN1A, CDKN2A, CDKN2D, and TP53 (Dodig et al. 2019; Herdy et al. 2022; Dehkordi et al. 2021; Kuilman et al. 2010; Cohn et al. 2023)—in astrocytes, microglia, oligodendrocytes, OPCs, and vasculature cells (Figure 1A–F). The gene count was normalized to ensure each cell had an equivalent total expression level (Figure 1B). These findings indicate that CDKN1A was widely expressed in all five cell types of interest. In contrast, CDKN2A shows less preferential expression in vascular cells; it is more highly expressed in oligodendrocytes and OPCs, whereas TP53 is mainly expressed in astrocytes. CDKN2D was less preferentially expressed because it has been reported to be mostly neuronal (Dehkordi et al. 2021). To investigate the pathological association between cellular senescence and AD, we compared the proportion of cells with gene counts > 0 for each canonical marker across cell types with amyloid density measurements (Figure 1G). These results demonstrate that CDKN2A annotates amyloid‐related senescent microglia, oligodendrocytes, and OPCs; TP53 annotates amyloid‐associated microglia. Senescent astrocytes and vasculature cells are not associated with amyloid density by these markers. Additionally, we found that the numbers of senescent microglia, oligodendrocytes, and OPCs are correlated at the subject level, indicating potential cell–cell interactions (Figure 1H). Taken together, cellular senescence markers are expressed heterogeneously in the brain, and CDKN2A predominantly annotates senescent microglia, oligodendrocytes, and OPCs. Based on these findings, it is plausible that stressed oligodendrocytes or OPCs are producing damaged myelin debris that drive microglial senescence.

## 3 AD Is Strongly Associated With Cellular Senescence and Cholesterol Dysregulation in Microglia

**FIGURE 2:**
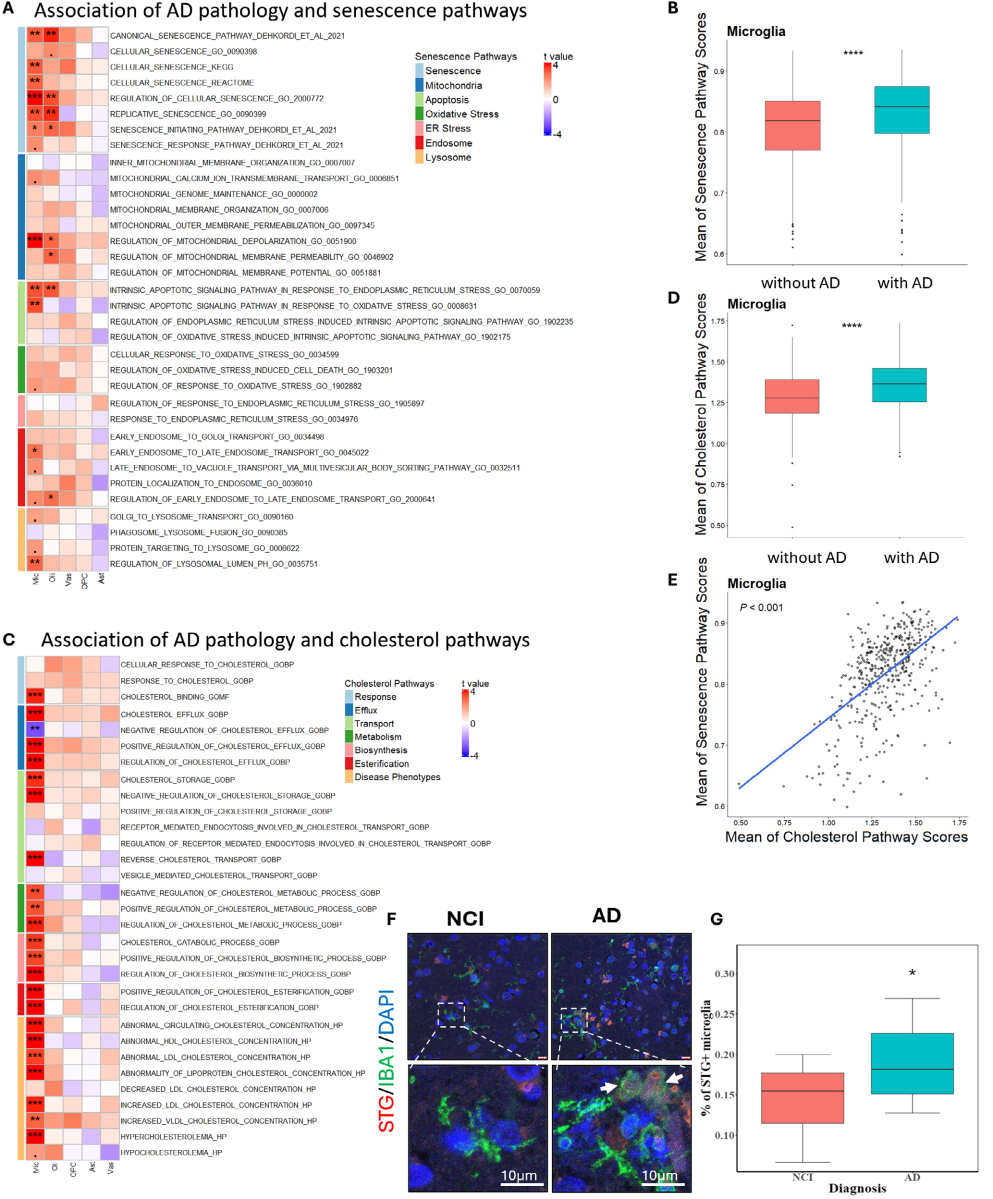
Microglia senescence is important in AD and is associated with cholesterol dysregulation. (A) Association of senescence related pathway scores with AD among different cell types using linear mixed effect model. The heatmap represents the t value which is the signed effect size divided by standard error. Significance is defined by “fdr” adjusted *p* value (.*p* < 0.1, *p < 0.05, **p < 0.01, ***p < 0.001). (B) The individual‐level mean of senescence pathway scores is calculated using the average of microglia “Senescence” (the light blue “Senescence” module in the panel 2A) pathway scores for each individual. (C) Association of cholesterol related pathway scores with AD among different cell types using linear mixed effect model. The heatmap represents the t value, which is the signed effect size divided by standard error. (D) The individual‐level mean of cholesterol pathway scores is calculated using the average of microglia cholesterol‐related (all pathways in the panel 2C) pathway scores together for each individual. Data shown are median ± quartiles and were analyzed using the Wilcoxon signed‐rank test (*p < 0.05, **p < 0.01, ***p < 0.001, ****p < 0.0001). (E) The association of senescence and cholesterol‐related pathway scores, calculated in panel 2B&2D. (F) Representative confocal immunofluorescence images of middle lobe sections stained for STG (senescence marker, red), IBA1 (microglial marker, green), and DAPI (nuclear marker, blue). Top panels show low‐magnification images, with dashed boxes highlighting areas of interest (bottom panels). White arrows show colocalization of STG and IBA1 in microglia. (G) Quantification of the percentage of STG+ IBA1+ microglia in AD and NCI. Linear mixed effect model: % positive ~ Diagnosis+(1|Individual) was used to properly account for the structure of the data (*n* = 20 ROIs: Each diagnosis group has 4 individuals and 5 ROIs per individual). Significance is defined by *p* value (*p < 0.05, **p < 0.01, ***p < 0.001, ****p < 0.0001). Box plots in inset show lower and upper hinges at the 25th and 75th percentiles, with whiskers extending to, at most, 1 times the interquartile range (IQR).

To comprehensively understand the association between AD and cellular senescence given the high‐dimensional nature of snRNA‐seq data, we investigated senescence‐related pathways—including mitochondrial function, ER stress, oxidative stress, apoptotic signaling, and endosome–lysosome trafficking—each of which is altered in the AD brain (Jagtap et al. 2023; Butterfield and Halliwell 2019). We calculated pathway enrichment scores for each cell using transcriptome‐wide profiles and tested whether AD pathology is associated with changes in senescence and related pathways across different non‐neuronal cell types. The results demonstrate that microglia and oligodendrocytes are susceptible to AD‐associated cellular senescence and its destructive biological processes, while vascular cells, OPCs, and astrocytes show less association with AD‐related senescence (Figure 2A). In microglia and oligodendrocytes, AD was associated with cellular senescence, mitochondrial abnormalities, and altered apoptotic signaling (Figure 2A). Specifically, the microglial endosome–lysosome system is markedly altered in AD, indicating the importance of microglial proteinopathy in exacerbating AD pathologies. Overall, microglia from AD patients showed a significantly higher pathway score for cellular senescence than microglia from control subjects (Figure 2B).

Because microglia play important roles in cholesterol homeostasis and myelin phagocytosis—and these functions are impaired in aged or AD subjects (Haney et al. 2024; McNamara et al. 2023; Byrns et al. 2024)—we hypothesized that senescent microglia have impaired cholesterol‐regulation machinery, contributing to worsened AD pathology. To test this, we examined whether microglia from AD patients (Figure 2C) exhibit dysregulation of cholesterol‐related pathways—including cholesterol response, efflux, storage, transport, metabolism, biosynthesis, esterification, lipid particles, and abnormal cholesterol levels—as seen in senescent microglia (Figure S1). We found that AD microglia and senescent microglia (defined as cells with a senescence pathway score>mean + 2× standard deviation) share similar alterations across the spectrum of cholesterol‐related pathways (Figure 2C; Figure S1). Overall, microglia from AD participants showed a significantly higher cholesterol‐related pathway score than control microglia (Figure 2D), and cholesterol scores were highly correlated with senescence scores (Figure 2E). STG staining and co‐immunostaining of human cortex slides showed that AD patients had higher levels of STG in microglia (IBA1+ cells), indicating that AD is associated with increased senescent microglia (Figure 2F,G).

## 4 2.3Specific Microglial States Are Susceptible to Cholesterol‐Associated Cellular Senescence in AD

Microglia are highly dynamic and display a range of activation states—including homeostatic, inflammatory, phagocytic, and lipid‐processing—each of which can be affected by AD. The susceptibility of these states to senescence in AD, as well as the molecular pathways driving their transitions, remains a critical area of investigation. To unveil state‐specific alterations in cellular senescence and cholesterol‐related processes during early and late AD, we reanalyzed the snRNA‐seq profile of 164,076 microglial nuclei from six regions (prefrontal cortex, hippocampus, mid‐temporal cortex, angular gyrus, entorhinal cortex, and thalamus) of postmortem brain tissue from 424 participants (Table S2) in ROSMAP. Microglia were classified into 12 clusters—MG0: homeostatic; MG1: neuronal surveillance; MG2: inflammatory I; MG3: ribosome biogenesis; MG4: lipid processing; MG5: phagocytic; MG6: stress‐related; MG7: glycolytic; MG8: inflammatory II; MG10: inflammatory III; MG11: antiviral; and MG12: cycling—based on signatures from the original publication (Sun et al. 2023).

**FIGURE 3:**
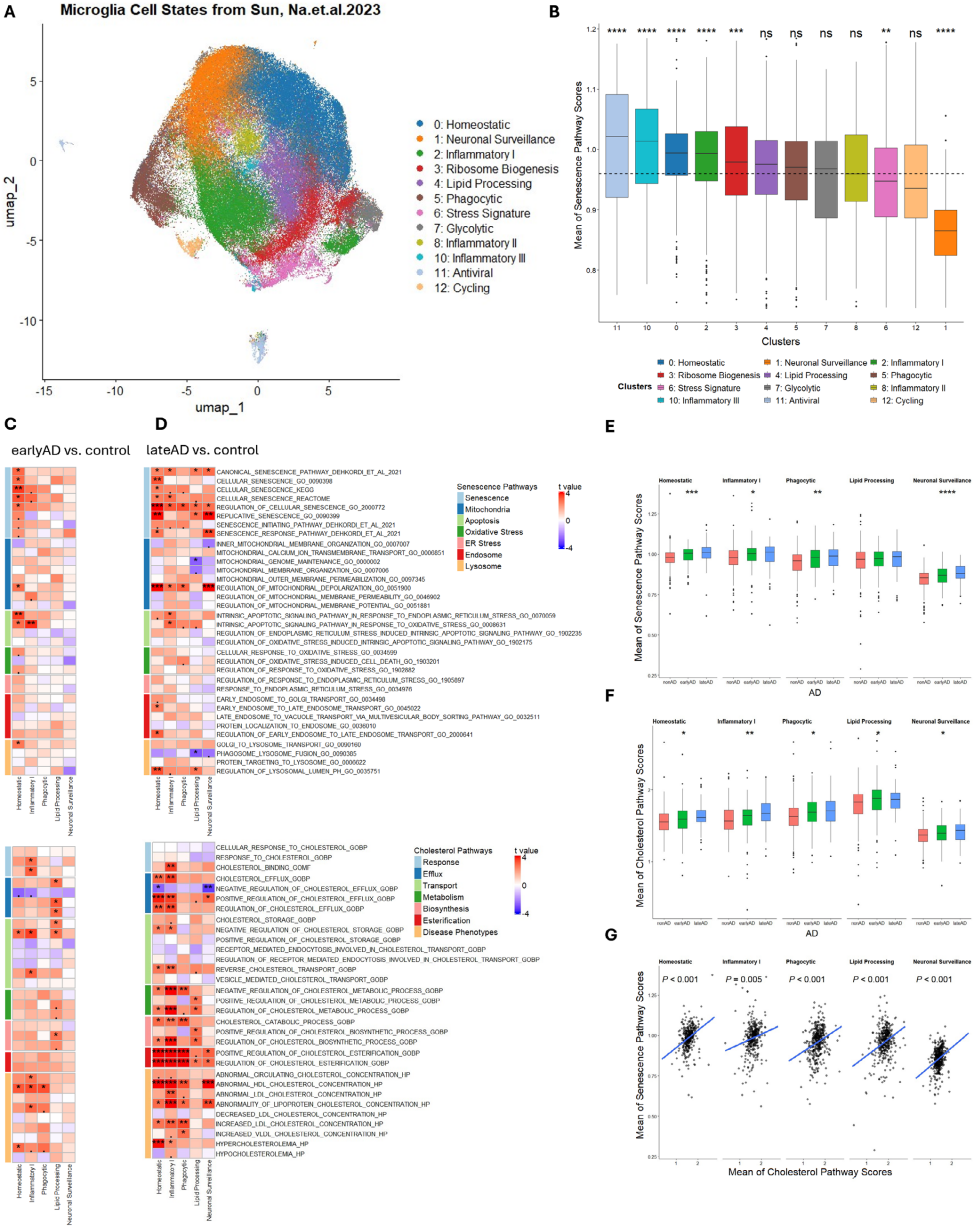
Cell states of microglia are susceptible to cholesterol‐related senescence in AD. (A) UMAP of 164,076 microglia nuclei with annotated microglial states in ROSMAP 6‐region snRNA‐seq data. (B) The individual‐level mean of senescence related pathway scores among each microglia cell state calculated using the same method as Figure 2B. (C, D) Association of senescence and cholesterol related pathway scores with AD disease stages (C: EarlyAD vs. control; D: LateAD vs. control) in different microglia cell states using linear mixed effect model. The heatmap represents the t value which is the signed effect size divided by standard error. Significance is defined by “fdr” adjusted *p* value (.*p* < 0.1, *p < 0.05, **p < 0.01, ***p < 0.001). (E, F) Summaries of the individual‐level mean of senescence and cholesterol related pathway scores across AD disease stages in C,D. Data shown are median ± quartiles and were analyzed using the Kruskal–Wallis test (*p < 0.05, **p < 0.01, ***p < 0.001, ****p < 0.0001). (G) The association of senescence and cholesterol related pathway scores in homeostatic, inflammatory I, phagocytic, lipid processing, and neuronal surveillance microglia.

To determine which states are susceptible to senescence, we compared senescence scores across these clusters. Antiviral microglia exhibited the highest scores, whereas neuronal‐surveillance microglia exhibited the lowest (Figure 3A,B). We then assessed how each state's score changed in early versus late AD (Figure 3C,D). Homeostatic and inflammatory states increased in early AD, while homeostatic, inflammatory, phagocytic, lipid‐processing, and neuronal‐surveillance states acquired senescence features in late AD. Consequently, these five states displayed significantly higher senescence pathway scores (Figure 3E).

Next, we evaluated cholesterol homeostasis scores by state across AD stages. In early AD, inflammatory, lipid‐processing, and homeostatic microglia exhibited altered cholesterol‐related pathways; in late AD, homeostatic, inflammatory, phagocytic, lipid‐processing, and neuronal‐surveillance states showed impaired cholesterol homeostasis (Figure 3C,D). These same five states also had significantly higher cholesterol‐related pathway scores (Figure 3F), and cholesterol scores were highly correlated with senescence scores (Figure 3G). These findings raise the possibility that cholesterol dysregulation may drive cellular senescence in specific microglial states as AD progresses. In early AD, homeostatic and inflammatory states are particularly vulnerable, while in late AD, key functions—lipid processing, phagocytosis, and neuronal surveillance—are disrupted by cholesterol‐induced senescence, exacerbating pathology.

## 5 Inflammatory Microglia Dominantly Drive Cholesterol‐Associated Cellular Senescence Signatures in SEA‐AD

**FIGURE 4:**
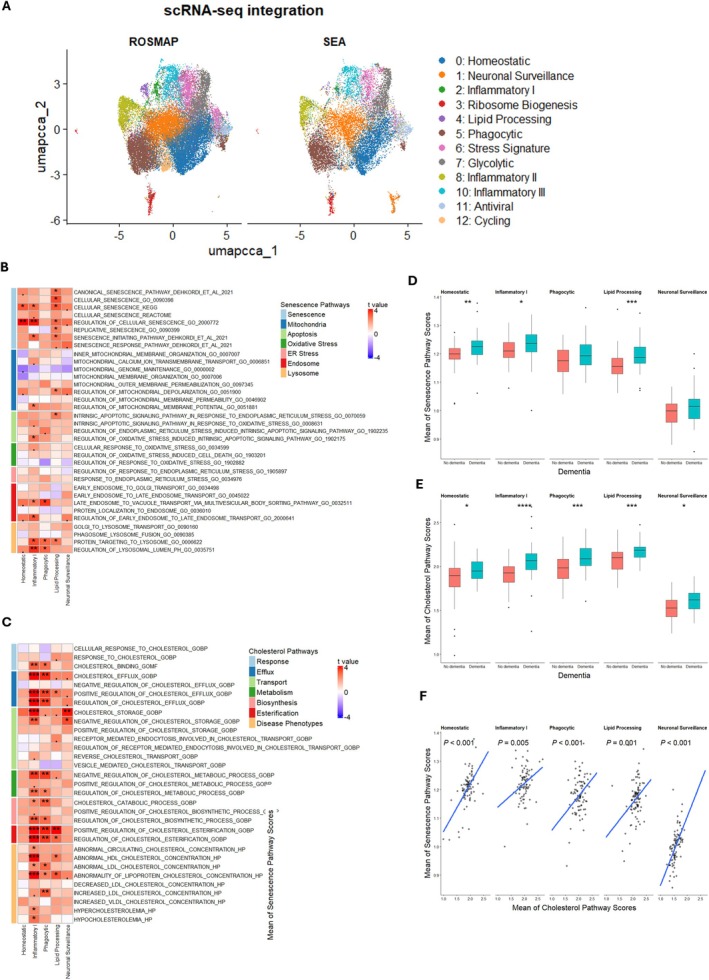
Cell states of microglia from SEA‐AD are susceptible to cholesterol‐related senescence in dementia. (A) UMAP of microglia nuclei with annotated microglial states in SEA‐AD snRNA‐seq data projection from microglia cell states in Figure 3. (B, C) Association of senescence and cholesterol related pathway scores with dementia in homeostatic, inflammatory I, phagocytic, and lipid processing microglia using a linear mixed effect model. The heatmap represents the t value which is the signed effect size divided by standard error. Significance is defined by “fdr” adjusted *p* value (.*p* < 0.1, *p < 0.05, **p < 0.01, ***p < 0.001). (D, E) The individual level of senescence and cholesterol‐related pathway scores by dementia in homeostatic, Inflammatory I phagocytic and lipid processing microglia calculated using the same method as Figure 2B. Data shown are median ± quartiles and were analyzed using the Wilcoxon signed‐rank test (*p < 0.05, **p < 0.01, ***p < 0.001, ****p < 0.0001). (F) The association of senescence and cholesterol‐related pathway scores in homeostatic, inflammatory I, phagocytic, and lipid processing microglia.

Single‐cell RNA‐seq enables full transcriptional characterization of cellular senescence based on microglial cell states. However, it is challenging to compare without bias the association between cellular senescence and impaired cholesterol homeostasis in AD development across datasets, owing to variance from assay methods, technical effects, and biological backgrounds. To improve the robustness of our hypothesis on senescence–cholesterol associations in AD microglial states, we used Seurat's embedded CCA Integration function to project microglia from SEA‐AD (Seattle Alzheimer's Disease Brain Cell Atlas) (Table S3; Figure 4A) onto the cell states defined in ROSMAP (Figure 3A), accounting for experimental and biological factors.

We then compared senescence‐related and cholesterol‐related pathway enrichment scores between clinically diagnosed dementia and non‐dementia patients in homeostatic, inflammatory, phagocytic, and lipid‐processing microglia (Figure 4B–C). These analyses demonstrate that inflammatory microglia are susceptible to cellular senescence and cholesterol pathway dysregulation in dementia, whereas senescence‐ and cholesterol‐related pathways are not significantly altered in homeostatic microglia when examined separately.

Next, we summarized both senescence‐related and cholesterol‐related pathway enrichment scores in homeostatic and inflammatory microglia, finding that both scores increase with dementia development (Figure 4D–E) and that cholesterol scores correlate strongly with senescence scores (Figure 4F). This cholesterol‐related cellular senescence in inflammatory microglia may contribute to loss of microglial functions such as phagocytosis and to the emergence of neurodegenerative pathology phenotypes such as amyloid plaques or tangles.

## 6 2.5Cholesterol‐Associated Senescence Transcriptomic Signatures Obtained Using ROSMAP snRNA‐Seq Data in Microglia

**FIGURE 5:**
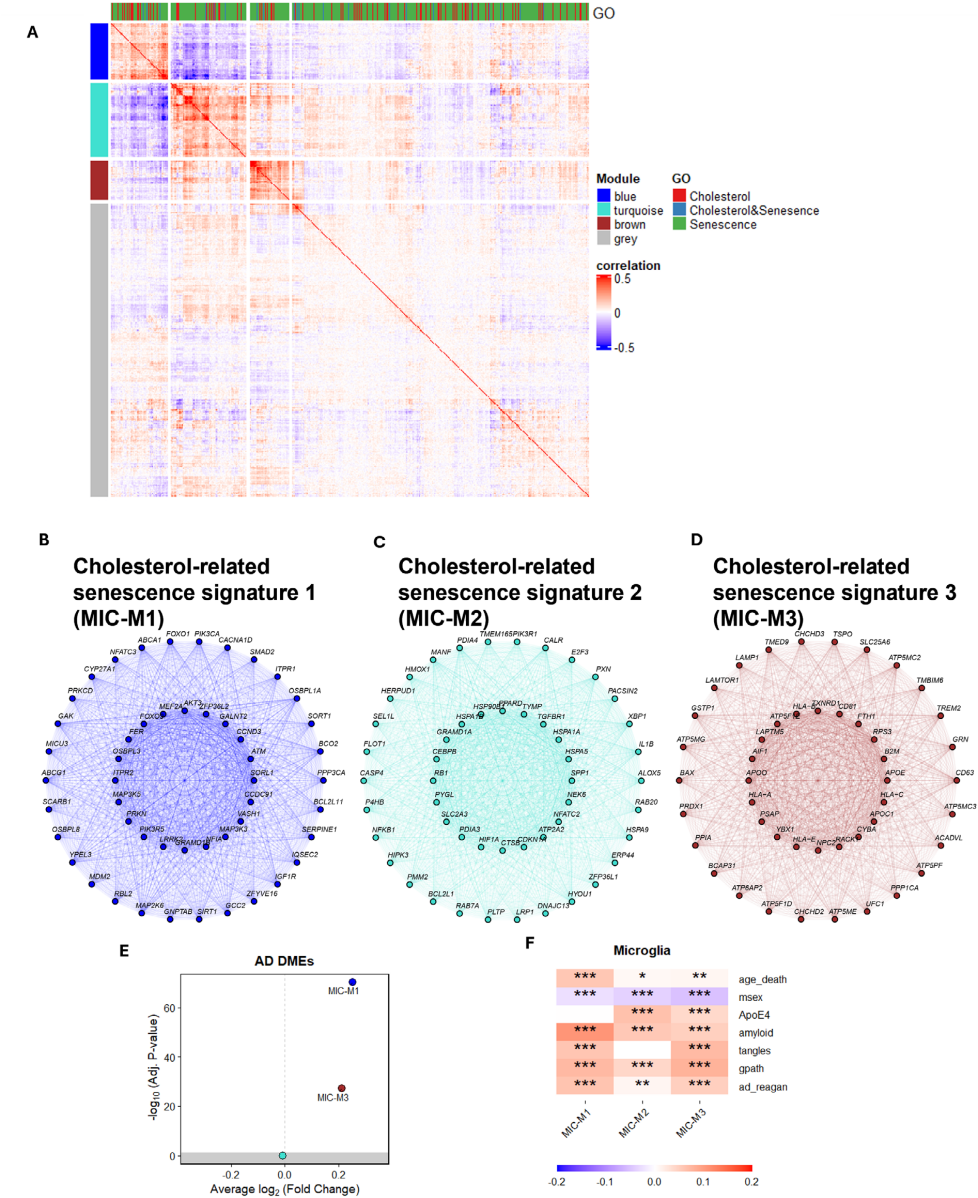
WGCNA gene correlation network analysis shows modules containing senescence and cholesterol‐related genes in human microglia. (A) Genes related to senescence and cholesterol are selected for gene correlation network analysis. The heatmap represents the gene–gene correlation, and their module identities are labeled blue, turquoise, and brown (gray means not correlated) in human microglia (data from Figure 2). (B–D) Diagrams of the three modules with 20 hub genes and 30 remaining genes. These marker genes represent the cholesterol‐related senescence signatures for the following results in Figure 6B,C. (E) Differential expression of module eigengenes by AD. (F) Module‐trait association plots between the three modules and AD‐related traits.

To further increase our confidence, we employed co‐expression network analysis to dissect the interplay between cholesterol dysregulation and senescence, enabling the identification of gene modules and key regulatory hubs involved in cholesterol‐related senescence pathways in microglia. We compiled a list of cholesterol‐ and senescence‐related genes from the Gene Ontology (GO) database and performed weighted gene co‐expression network analysis (WGCNA) on the ROSMAP dataset to retrieve co‐expression modules representing cholesterol‐associated senescence transcriptomic signatures (Figure 5A). We identified three co‐expression modules with altered disease‐related characteristics in vivo (Figure 5B–D).

Module 1 comprises central regulators of lipid metabolism, stress resistance, and cellular senescence—APOE, FOXO3, SORL1, ATM, and CDKN1A (p21). APOE4 is the strongest genetic risk factor for late‐onset AD, regulating cholesterol transport and amyloid‐β pathology. SORL1 prevents amyloid accumulation; ATM and CDKN1A link DNA damage, senescence, and inflammation to microglial dysfunction in AD; and FOXO3 underscores microglial stress resistance and neuroprotection under senescence‐inducing conditions.

Module 2 includes HSPA1A (HSP70), HSP90, GRAMD1A, and ATP2A2, which emphasize protein homeostasis, cholesterol transport, and calcium regulation—processes critical to microglial function in AD. HSP70 and HSP90 act as molecular chaperones mitigating protein aggregation, while GRAMD1A and ATP2A2 implicate disruptions in cholesterol transport and calcium homeostasis that contribute to cellular stress, senescence, and inflammation.

Module 3 is enriched for cholesterol‐ and immune‐related genes—APOE, APOC1, HLA‐A, NPC2, AIF1 (IBA1), and PSAP. APOE and APOC1 reflect dysregulated lipid metabolism leading to lipid‐droplet accumulation, amyloid‐β deposition, and neuroinflammation. HLA‐A and AIF1 mark activated microglia that exacerbate neurodegeneration, while NPC2 and PSAP mediate lysosomal cholesterol trafficking and lipid metabolism, processes often impaired in AD microglia, resulting in cholesterol accumulation and cellular stress.

We tested whether these module eigengenes were differentially expressed by AD diagnosis (Figure 5E) using Seurat's ModuleEigengenes differential‐expression function and assessed their associations with AD‐related variables using the moduleTraitCor function (Morabito et al. 2023). Module 1 (blue) and Module 3 (brown) eigengenes were significantly upregulated in AD pathology samples (Figure 5E), and all three modules correlated with AD‐related traits (Figure 5F), indicating that cholesterol‐associated senescence signatures are altered in AD. Many genes in these modules—including APOE, SORL1, APOC1, and CDKN1A—are well‐established AD risk factors, reinforcing the link between cholesterol‐induced cellular senescence, microglial dysfunction, and neuroinflammation. Together, these modules integrate essential processes—cholesterol dysregulation, cellular senescence, and immune activation—within microglia, highlighting their critical role in AD pathogenesis.

## 7 Myelin and Oxysterols Induce DAMs and Cholesterol‐Related Senescence Signatures

Microglia can receive multiple lipid‐related stressors from damaged or aged oligodendrocytes, including cholesterol, oxysterols, phospholipids, ceramides, and sphingolipids (Loving and Bruce 2020). We previously found that oxysterols can induce brain senescence, and this phenotype can be rescued by cyclodextrins, cholesterol‐lowering molecules (Wang et al. 2025). iPSC‐derived microglia (iMGs) offer a versatile platform to investigate AD‐specific mechanisms in a controlled environment while maintaining relevance to human biology (Figure S2). These cells allow for functional assays and mechanistic studies to confirm the role of cholesterol dysregulation in driving microglial senescence and its relevance to AD pathology.

**FIGURE 6:**
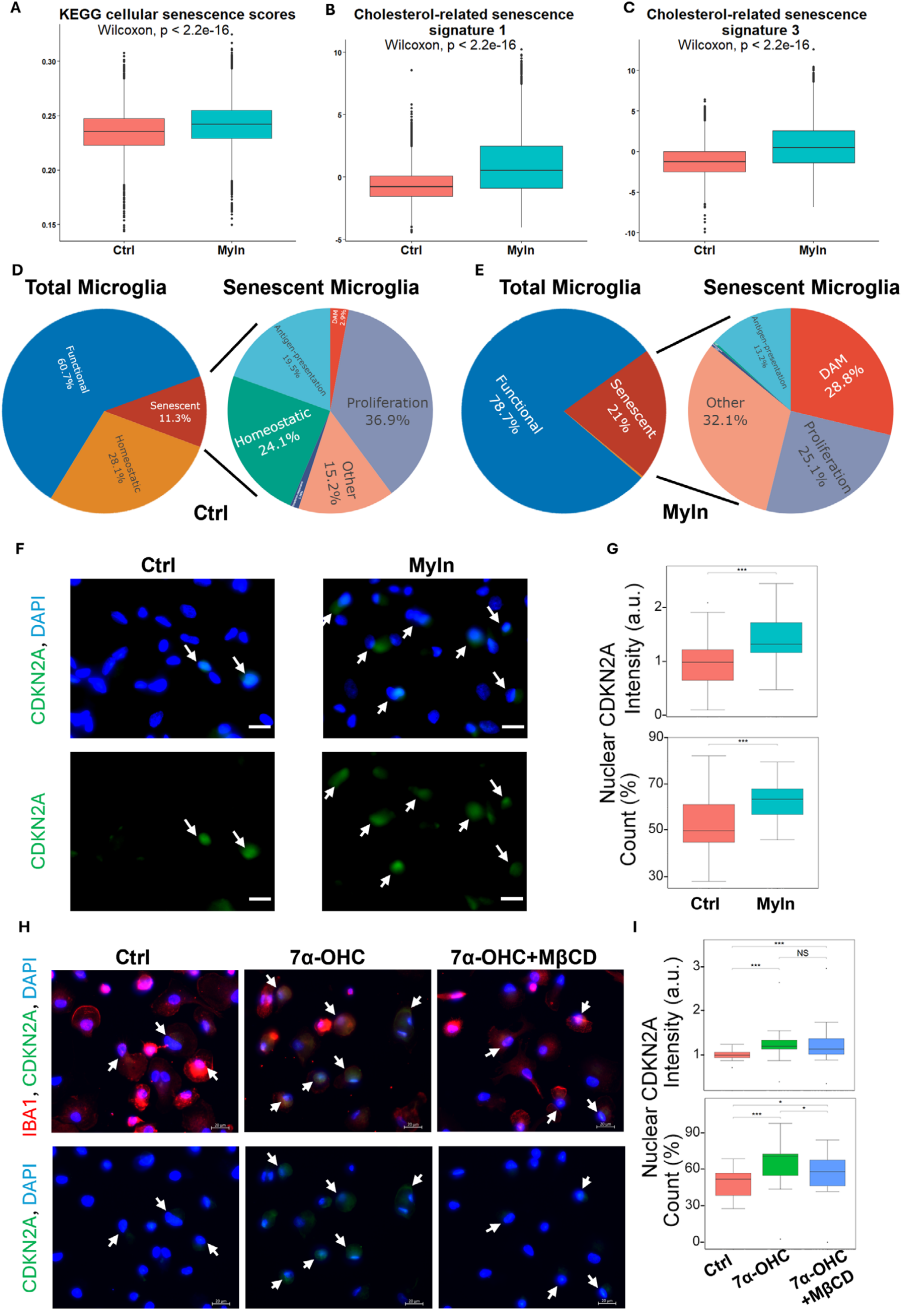
Myelin treatment induces DAM‐like signatures in iMG, displaying cholesterol‐related senescence. (A–C) Cellular senescence signatures (KEGG and cholesterol‐related senescence signatures from Figure 5B,D) after myelin treatments. Data shown are median ± quartiles and were analyzed using the Wilcoxon signed‐rank test. “Myln” means myelin treatment. “Ctrl” means control. (D, E) Percentage changes of cell state composition after myelin treatment in iMGs. Senescent state is identified for each cell if its senescence pathway enrichment score (KEGG in the A) is larger than the mean plus 1× standard deviation. (F) Representative images of CDKN2A (green) expression after myelin treatment for 24 h. DAPI (blue) marks the nuclei. Scale bars are 20 μm. (G) Quantification for nuclei intensity of CDKN2A and percentage of CDKN2A positive nuclei. Data shown are median ± quartiles and were analyzed using the Kruskal–Wallis test (*p < 0.05, **p < 0.01, ***p < 0.001). Box plots in inset show lower and upper hinges at the 25th and 75th percentiles, with whiskers extending to, at most, 1.5 times the interquartile range (IQR). (H) Representative images of CDKN2A (green) expression after 7α–OHC treatment for 24 h and rescue with MβCD. IBA1 (red) is a common microglia marker. DAPI (blue) marks the nuclei. Scale bars are 20 μm. (I) Quantification for nuclei intensity of CDKN2A and percentage of CDKN2A positive nuclei. Data shown are median ± quartiles and were analyzed using the Kruskal–Wallis test (*p < 0.05, **p < 0.01, ***p < 0.001). Box plots in inset show lower and upper hinges at the 25th and 75th percentiles, with whiskers extending to, at most, 1.5 times the interquartile range (IQR).

We reanalyzed snRNA‐seq data from 56,454 iMGs treated for 24 h with four pathological CNS substrates—synaptosomes (Syn), myelin debris (Myln), apoptotic neurons (Apop), and synthetic amyloid‐β (Ab) fibrils (Dolan et al. 2023). These AD‐related substrates yielded distinct clusters of disease‐associated microglia (DAMs; Cluster 2 and Cluster 8) (Figure S3A). Treating iMGs with myelin increased KEGG cellular senescence scores (Figure 6A). DAMs—defined by ABCA1, APOE, GPNMB, and TREM2 signatures—exhibited significant perturbation of cholesterol‐related bioprocesses after myelin exposure, linking these cells to lipid handling and disease‐associated pathways (Figure S3B–C,J).

We next applied the three cholesterol‐related senescence signatures (Modules 1–3; Figure 5B–D) derived from human brain microglia to the iMG dataset. Signatures 1 and 3 (Modules 1 & 3) showed increased expression after myelin treatment (Figure 6B,C). Among the four pathological CNS substrates—Syn, Myln, Apop, and Ab—myelin is the strongest inducer of cholesterol‐related senescence signatures in DAMs (Figure S3D–I). These transcriptomic states mirror cholesterol‐related senescence in human AD microglia.

Analysis of cellular‐state composition revealed that myelin treatment increased the proportion of senescent microglia from 11.3% to 21.0% (Figure 6D,E). Among senescent cells, DAMs rose from 2.9% to 28.8% post‐treatment, and DAM/inflammatory signature gene expression (ABCA1, APOE, GPNMB, TREM2) also increased significantly (Figure S3J,K). Together, these data support a model in which myelin debris drives iMGs toward a DAM‐like state with cholesterol‐related senescence features (Figure 5).

To validate these findings with a canonical senescence marker, we treated iMGs with myelin and observed elevated nuclear expression and an increased percentage of CDKN2A^+^ cells (Figure 6F,G). Changes in nuclear morphology—specifically increased nuclear area and decreased eccentricity—are established markers of senescence, reflecting loss of nuclear integrity. Myelin treatment induced significant alterations in these features (Figure S4).

Because myelin is cholesterol‐rich, we hypothesized that oxysterol derivatives drive senescence. We treated iMGs with 7α‐oxocholesterol (7α‐OHC) and observed increased microglial senescence, which was reversed by methyl‐β‐cyclodextrin (MβCD) treatment, as measured by reduction in CDKN2A^+^ cells (Figure 6H,I). These findings underscore the critical role of cholesterol overload in driving microglial senescence and its potential contribution to AD pathogenesis.

## 8 Discussion

In this study, we integrated four independent single‐cell transcriptomic datasets to identify AD‐associated senescence markers, gene expression differences, biological pathways, and co‐expression modules. We annotated the diversity of cell types and cell states within AD‐associated senescence molecular signatures at both single‐cell and pseudo‐bulk levels. Using integrative analysis techniques, we examined preserved biological information on senescence signatures and microglial cell states across different datasets (ROSMAP and SEA‐AD) and models (human postmortem biospecimens and iPSC‐derived microglia). We focused on AD‐associated senescence signatures across major non‐neuronal cell types in the central nervous system (CNS) and found that microglia from subjects with AD exhibit highly perturbed senescence signatures. This suggests that microglial dysfunction is central to the pathology of AD‐related senescence. Further analysis showed that specific microglial states—homeostatic, inflammatory, phagocytic, lipid‐processing, and neuronal‐surveillance—are particularly vulnerable to AD‐associated senescence. Among these, the inflammatory microglial state dominates the effects of senescence in early AD, highlighting microglia as the most susceptible cell type to AD‐related senescence.

We examined senescence‐related pathways—including mitochondrial dysfunction, ER stress, oxidative stress, apoptotic signaling, and endosome–lysosome trafficking (Rawji et al. 2016). Distinct processes were observed, such as mitochondrial depolarization, stress‐induced apoptosis, early‐to‐late endosome transport, and lysosomal pH imbalance (Fancy et al. 2024). A comparison of molecular perturbations between microglia from AD subjects and senescent microglia revealed that cholesterol‐related bioprocesses were highly dysregulated in both groups, suggesting that cholesterol dysregulation is a key driver of AD‐associated senescence pathologies (Wang et al. 2025). More specifically, our results highlight the importance of microglial cholesterol homeostasis in AD pathology and suggest that targeting cholesterol metabolism in senescent microglia could be a promising therapeutic strategy (Haney et al. 2023). The identification of specific microglial states that are more vulnerable to senescence and cholesterol dysregulation provides a clearer understanding of the cell‐type and state‐specific mechanisms driving AD progression (Sun et al. 2023). This opens the door to potential interventions aimed at modulating microglial function and cholesterol homeostasis to mitigate neurodegeneration in AD.

Microglia exhibit remarkable plasticity, transitioning between pro‐inflammatory and anti‐inflammatory states to maintain homeostasis and respond to injury or disease (Isik et al. 2023; Liu et al. 2023). However, during aging and disease progression, microglial function becomes dysregulated, potentially amplifying neuroinflammatory responses and contributing to synaptic loss and neuronal damage. While microglial senescence has been observed in AD, its precise mechanisms and impact on disease progression—particularly across the diverse functional states these cells adopt—remain incompletely understood. Understanding the contributions of senescence in microglia and other cell populations is crucial for unraveling the complex cellular interactions that drive AD pathology.

Cholesterol dysregulation plays a critical role in microglial dysfunction in AD (Chang et al. 2017; Muñoz Herrera and Zivkovic 2022). Cellular metabolism, including lipid metabolism, is intricately linked to the regulation of cellular health and aging processes (Ziegler et al. 2024). Cholesterol homeostasis—a subset of lipid metabolism—is particularly essential for microglial function due to its role in maintaining membrane integrity, signal transduction, and lipid‐raft formation (Chen et al. 2022). In AD, disruptions in cholesterol metabolism—including altered synthesis, efflux, and trafficking—lead to the accumulation of toxic cholesterol intermediates and lipids (Wang et al. 2025). Excess cholesterol or its oxidized derivatives, such as oxysterols, can act as potent stressors, triggering oxidative stress and endoplasmic reticulum (ER) stress (Wang et al. 2025; de Medina et al. 2022). Aged or damaged oligodendrocytes and OPCs produce excessive myelin debris enriched in oxidized stressors. These responses are central to initiating cellular senescence—a state characterized by irreversible cell‐cycle arrest and the secretion of pro‐inflammatory factors (Butterfield and Halliwell 2019; Nousis et al. 2023). In the AD brain, interactions with amyloid‐β and tau pathology further disrupt cholesterol homeostasis and accelerate microglial dysfunction and senescence (Rudajev and Novotny 2022). These dynamics underscore the pivotal role of lipid metabolism—particularly cholesterol regulation—in cellular aging and highlight potential therapeutic avenues targeting these pathways to mitigate microglial senescence in AD.

A key finding of our study is that microglial senescence acts as the effector of cholesterol dysregulation in AD. Although astrocytes are the primary source of brain cholesterol, the role of microglia in mediating damage from cholesterol dysregulation is underappreciated. We showed that AD subjects exhibit alterations across multiple cholesterol‐related pathways—including efflux, storage, metabolism, and biosynthesis—in senescent microglia. This dysregulation is particularly pronounced in inflammatory and homeostatic microglial states. We observed a strong correlation between cholesterol dysregulation and microglial senescence, indicating that senescence exacerbates inflammation and microglial dysfunction in AD. These findings were further validated using data from iMGs treated with CNS substrates, where cholesterol‐associated senescence signatures were significantly altered. We identified three distinct gene co‐expression network modules representing cholesterol‐associated senescence signatures in AD microglia. Module 1 includes AD risk genes ABCA1, ABCG1, and SORL1, suggesting altered responses to amyloid‐β (Mishra et al. 2025). Module 2 contains inflammation‐related genes IL1B, NFKB1, and CEBPB, highlighting the importance of inflammatory states in AD‐associated senescence (Li et al. 2023). Module 3 comprises DAM genes APOE, B2M, and TREM2, indicating that senescent microglia are key contributors to AD pathology (Xu et al. 2022). These modules are also influenced by CNS substrate treatment in iMGs, further supporting the role of cholesterol‐associated senescence in driving AD progression. However, no single gene suffices to define senescence across brain cell types in scRNA‐seq data, likely due to limited sequencing depth and high dropout rates (Zyla et al. 2023). AD‐associated senescence is heterogeneous within and across cell types; thus, multiple markers and features should be considered in future studies (Cohn et al. 2023).

In conclusion, our study demonstrates that microglia play a central role in AD‐associated cellular senescence, with cholesterol dysregulation acting as a key driver of this process. Future research should focus on developing therapies that target specific microglial states and their associated cholesterol pathways to slow AD progression.

## 9 Methods

### 9.1 Database

Processed single‐nucleus transcriptomic data and ROSMAP metadata were downloaded from the Synapse AD Knowledge Portal (https://www.synapse.org/#!Synapse:syn52293417) with Synapse ID: syn52293433 (Figures 1, 2 and 5) and syn52383412 (Figure 3). Source data were collected from 427 (syn52293433) and 443 (syn52383412) subjects from ROSMAP (Mathys et al. 2023; Sun et al. 2023; De Jager et al. 2018). Nuclei were isolated from frozen postmortem brain tissues and subjected to droplet‐based single‐nucleus RNA sequencing (snRNA‐seq). Cell types were assigned based on Leiden clustering, marker gene analysis, and comparisons with previously published data in the original publication (Mathys et al. 2023; Sun et al. 2023).

Processed single‐nucleus transcriptomic data were downloaded from the Seattle Alzheimer's Disease Brain Cell Atlas (SEA‐AD) (Gabitto et al. 2024). Source data were collected from 89 participants (84 subjects and 5 references). Cell types were assigned by hierarchical probabilistic Bayesian mapping to BICCN reference in the original publication (Gabitto et al. 2024). Cell Ranger outputs of iMG (control and CNS substrate treatment) scRNA‐seq data were downloaded from Terra. Summary level data are available at https://app.terra.bio/#workspaces/Stevenslab/public_iMGLdatasets.

### 9.2 Differential Gene Expression (DGE) Analysis Using NEBULA

To perform DGE analysis, we utilized the NEBULA package available in R, which implements a negative binomial mixed‐effect model (He et al. 2021). The model is built to simultaneously account for overdispersion in RNA‐seq data and the correlation structure within clusters, ensuring that differentially expressed genes are identified accurately. To annotate senescence‐related genes, the keyword “SENESCENCE” was used on MsigDB (Molecular Signatures Database) gene sets C2 and C5.

### 9.3 Gene Set Enrichment Analysis (GSEA, Ucell) for Pathway Scores

The R package GSVA (method option “ssGSEA”) was used to calculate the pathway enrichment score (REACTOME_CELLULAR_SENESCENCE (M27188)) of each cell as the normalized difference in empirical cumulative distribution functions (CDFs) of gene expression ranks (Hänzelmann et al. 2013; Barbie et al. 2009). The cells were classified as senescent (Figure 2C: “SEN”) if their senescence pathway enrichment score is larger than the mean plus 2× standard deviation. The R package “UCell” was used to calculate the pathway enrichment scores for senescence‐related and cholesterol‐related pathways (Andreatta and Carmona 2021). UCell calculates gene signature scores for snRNA‐seq data based on the Mann–Whitney U statistic. UCell generates pathway enrichment scores based on gene rankings for each individual cell. The list of senescence‐related pathways was adapted from a study that used single nuclear transcriptomics to characterize senescent cells in AD (Fancy et al. 2024). The cholesterol‐related pathway list was generated from MsigDB using “cholesterol” as the keyword.

### 9.4 Microglia State Projection

The R package “Seurat” embedded “CCA‐Integration” function was used to project the microglia states in ROSMAP onto microglia in Sea‐AD (Stuart et al. 2019). Briefly, ROSMAP microglia were set as the reference dataset with predetermined cell state labels, and SEA‐AD microglia were set as the query dataset. Both datasets were log‐transformed first, and the top 2000 features were selected before the projection. After several iterations to test the query method for projection, the parameters (reduction = “pcaproject,” reference.reduction = “integrated.cca”, project.query = T) were used to predict microglia states in SEA‐AD from ROSMAP microglia states.

### 9.5 4Co‐Expression Network Analysis

We calculated the Pearson correlation coefficient between each pair of genes (including senescence‐related genes and cholesterol‐related genes) across individuals to generate a correlation matrix (*p*‐value < 0.05 in R) using microglia transcriptomic data from Figure 2. We constructed a co‐expression network and extracted the co‐expression modules using hdWGCNA from “Seurat” (Morabito et al. 2023). Module eigengene differential expression (DME), connectivity, and module‐trait association were calculated using functions provided by hdWGCNA (Morabito et al. 2023).

### 9.6 SenTraGor (STG) Staining

Formalin‐fixed paraffin‐embedded (FFPE) human brain slides containing the middle frontal lobe region were deparaffinized using xylene (3 min each, twice), xylene:ethanol (1:1, 3 min each, twice), 100% ethanol (3 min each, twice), 95% ethanol (3 min each, twice), 70% ethanol (3 min each, twice), and 50% ethanol (3 min each, twice), followed by rinsing in cold running water for 5 min. Antigen retrieval was performed by incubating slides in sodium citrate buffer (10 mM sodium citrate, 0.05% Tween 20, pH 6.0) for 30 min. Slides were washed with TBS for 5 min and incubated with 2 mM SenTraGor (STG, Bio‐Techne, #7555, dissolved in 100% ethanol, filtered with a 0.45 μm filter) for 10 min at room temperature. After incubation, slides were washed twice with 50% ethanol and once with TBST (0.025% Triton X‐100 in TBS) for 5 min each. Primary antibodies (goat anti‐biotin, anti‐ABCA1, and anti‐IBA1) were applied, and the slides were incubated overnight at 4°C. Following three TBST washes, slides were incubated with fluorescein‐conjugated secondary antibodies for 1 h at room temperature. Autofluorescence was quenched for 5 min, and the slides were washed three more times with TBST, mounted using F4680 mounting medium (Sigma), and imaged using a Zeiss Axio Scan slide scanner (Z1, 20× objective). Image analysis was performed with CellProfiler software.

### 9.7 Myelin and 7α‐OHC Treatment on iPSC Derived Microglia

The method of microglial differentiation was adapted from an existing protocol (Lanfer et al. 2022). To generate induced microglia cells (iMGs), human iPSCs were first differentiated into hematopoietic progenitor cells (HPCs) using a stepwise protocol. iPSCs were seeded at ~2 × 10^4^ cells/cm^2^ in mTeSR plus medium and transitioned to a hematopoietic differentiation medium on day 1, containing 50 ng/mL BMP4, 50 ng/mL VEGF, and 20 ng/mL SCF. Cells were maintained in this medium for 8 days, with sequential harvesting every 2 days to maximize HPC yield. Harvested cells were centrifuged at 300 x g for 5 min, resuspended in freezing medium, and stored for biobanking or used immediately for further differentiation. HPCs were then cultured in iMGL differentiation medium containing 50 ng/mL IL‐34, 100 ng/mL TGF‐β1, and 25 ng/mL M‐CSF to promote microglial maturation. The iMGLs were grown on uncoated glass coverslips to increase yield while preserving differentiation efficiency and functional characteristics.

Myelin was isolated from C57BL/6J mice as follows: mice were transcardially perfused with cold PBS, and the brains were manually homogenized in RPMI medium. The homogenate was layered onto a Percoll gradient and centrifuged at 500 x g for 30 min. The top layer, containing myelin, was collected and washed twice with distilled water to remove impurities. The purified myelin was then used in experiments at a concentration of 0.1, 1, and 10 μg/mL. Myelin and 7α‐OHC were prepared in FBS‐free complete medium, which contained all cytokines required for microglia differentiation. The microglia were treated with either myelin or 7α‐OHC for 24 h, followed by removal of the treatment medium and replacing it with MβCD prepared in FBS‐free complete medium if applicable.

### 9.8 Immunocytochemistry

iMG were cultured in 8‐well cell culture chamber slides (Bioland Scientific, 07–2108) with myelin treatment for 3 days. After fixation with 4% paraformaldehyde for 15 min, cells were permeabilized with 1% Triton X‐100 for 30 min and then blocked with 10% goat serum and 0.1% Triton X‐100 for 1 h at room temperature. Following blocking, nuclei were stained with Hoechst 33258 (Thermo Fisher, H3569) at a 1:12,000 dilution for 5 min. The slides were washed again and mounted with mounting medium (Sigma, F4680). Fluorescent images were captured using a Zeiss Axiovert 200 M Inverted Fluorescence Microscope with a 20× objective and analyzed using CellProfiler. Eccentricity is a shape measurement that quantifies how elongated or stretched an object is, approximating its shape to an ellipse. We used “CellProfiler” to fit an ellipse that best represented nuclear shape. Eccentricity was calculated as the ratio of the distance between the foci of the fitted ellipse and of the length of its major axis.

### 9.9 Statistics

The linear mixed effect model was used with AD pathology as the fixed effect and sample origin as the random effect (parameters and formula in the method) to test if the pathway score was associated with the variables of interest, using the following formula: pathway score ~ interested variables + size factor (library size of each cell) + (1|projid (sample id)). Multiple comparisons were corrected using the false discovery rate (FDR) method. A two‐tailed Student's *t*‐test or a one‐way ANOVA followed by a post hoc Tukey's test with *p* < 0.05 considered significant were used to test the AD effect.

## Author Contributions

Boyang Li designed the study, performed the experiments, analyzed the data, and wrote the manuscript. Shaowei Wang, Bilal Kerman, and Cristelle Hugo helped with the experiments on human tissues and iPSC‐microglia. E Keats Shwab, Chang Shu, and Ornit Chiba‐Falek reviewed the analysis of single‐nucleus RNA sequencing data. Zoe Arvanitakis facilitated access to single‐nucleus RNA sequencing data and brain samples. Hussein Yassine supervised the study design, data acquisition, data analysis, and the manuscript revision.

## Conflicts of Interest

The authors declare no conflicts of interest.

## Supporting information


**Figure S1:** Changes of cholesterol pathways in senescent microglia (SEN: senescent state is identified for each cell if its senescence pathway enrichment score is larger than the mean plus 2× standard deviation). Association of cholesterol related pathways scores with senescent states among microglia using linear mixed effect model. The heatmap represents the t value which is the signed effect size divided by standard errors. Significance is defined by “fdr” adjusted *p* value (.*p* < 0.1, *p < 0.05, **p < 0.01, ***p < 0.001).

**Figure S2:** Successful induction of iPSC‐derived microglia (iMG). (A–C) iMGs express common microglia markers IBA1 (red in A), TMEM119 (red in B) and TREM2 (red in C). DAPI marks the nuclei. iMGs generated from iPSCs. Scale bars 20 μm. (D) Percentage expression of IBA1 positive microglia in iMG from the results of Figure 6H,I

**Figure S3:** Cholesterol‐senescence signatures increase in microglia treated by myelin. (A) Two DAM clusters are identified after CNS substrate treatment. (B, C) The cell level of cholesterol pathway scores are calculated using the mean of microglia cholesterol‐related (the same set of pathways as Figure 2C) pathways scores together for each individual. Data shown are median ± quartiles and were analyzed using the Wilcoxon signed‐rank test. “Myln” means myelin treatment. “Ctrl” means control. (D–I) The expression levels of the module Eigen genes from Figure 5 in DAM cluster 2 & 8 after CNS substrate treatment. (J) Expression levels of DAM signature genes in the total microglia. (K) Expression levels of inflammatory signature genes in the total microglia.

**Figure S4:** Myelin treated iMGs show changed nuclear morphology. (A–D) Illustration of the nuclear morphology changes of senescent cells. (E, F) myelin treated iMG show decreased eccentricity and increased size. Linear mixed effect model: Variable ~ Treatment+1|Biological replicate, was used to properly account for the structure of the data (*n* = 18 ROIs: each treatment has 3 biological replicates and 6 technical replicates). Significance is defined by *p* value (*p < 0.05, **p < 0.01, ***p < 0.001). Box plots in inset show lower and upper hinges at the 25th and 75th percentiles, with whiskers extending to, at most, 1 times the interquartile range (IQR).

**Figure S5:** Illustration of the mechanism that myelin debris from oligodendrocytes causes cholesterol dysregulation in microglia, leading to senescent cell states in AD.

**Table S1:** ROSMAP participant characteristics for PFC snRNA‐seq data in Figures 1 and 2.

**Table S2:** ROSMAP participant characteristics for six‐region snRNA‐seq data in Figure 3.

**Table S3:** SEA‐AD participant characteristics for snRNA‐seq data in Figure 4.

## Data Availability

The data that support the findings of this study are available from the corresponding author, upon reasonable request.
