# Supplementary figures and images for: Microglial States Are Susceptible to Senescence and Cholesterol Dysregulation in Alzheimer's Disease
Source: Aging Cell. 2025 Aug 11;24(10):e70189. doi: 10.1111/acel.70189 (PMC12507412; doi:10.1111/acel.70189)

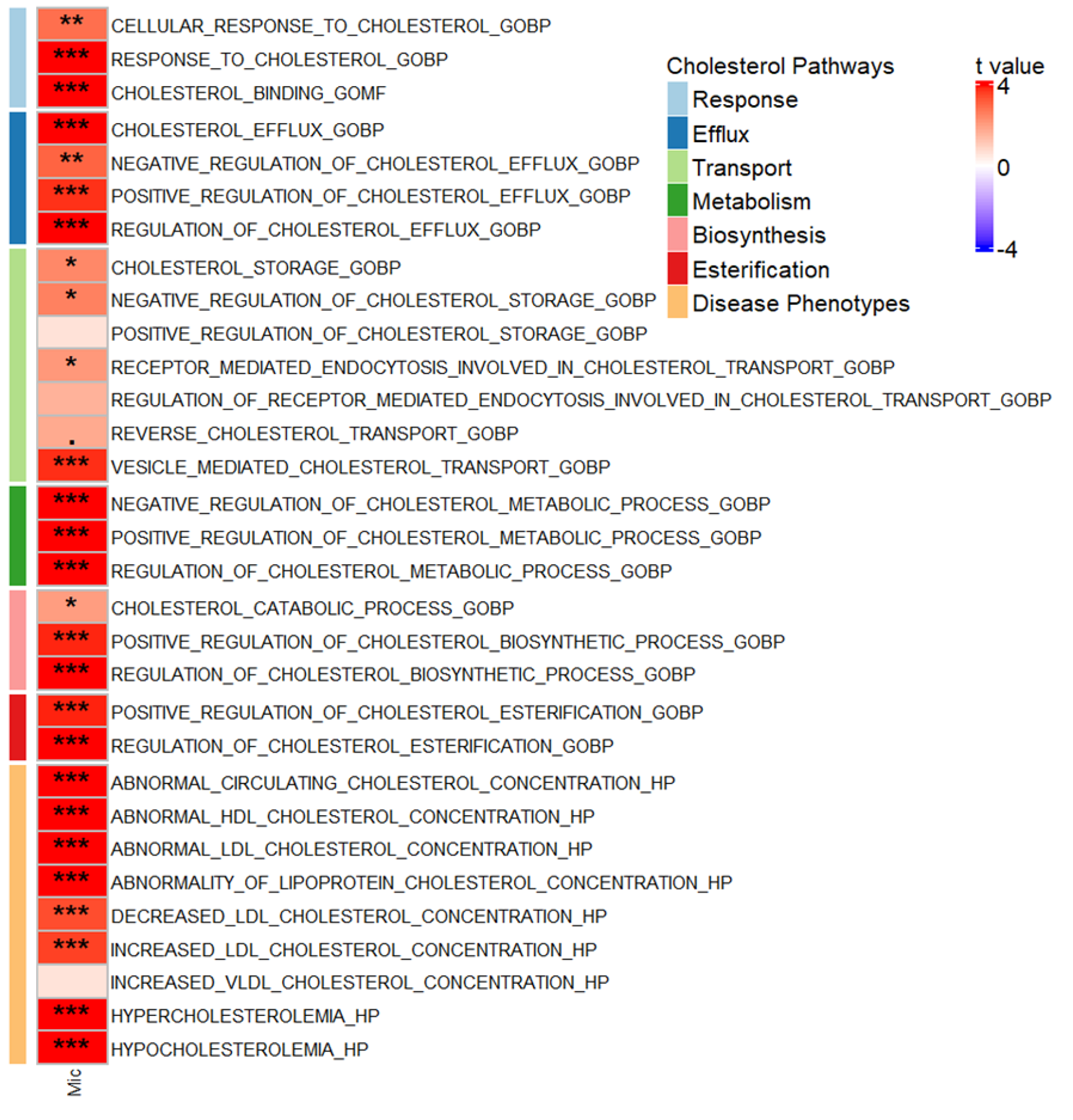

Supplement: Supplementary file 1 — Figure S1: Changes of cholesterol pathways in senescent microglia (SEN: senescent state is identified for each cell if its senescence pathway enrichment score is larger than the mean plus 2× standard deviation). Association of cholesterol related pathways scores with senescent states among microglia using linear mixed effect model. The heatmap represents the t value which is the signed effect size divided by standard errors. Significance is defined by “fdr” adjusted p value (.p < 0.1, *p < 0.05, **p < 0.01, ***p < 0.001). [file ACEL-24-e70189-s007.tif]

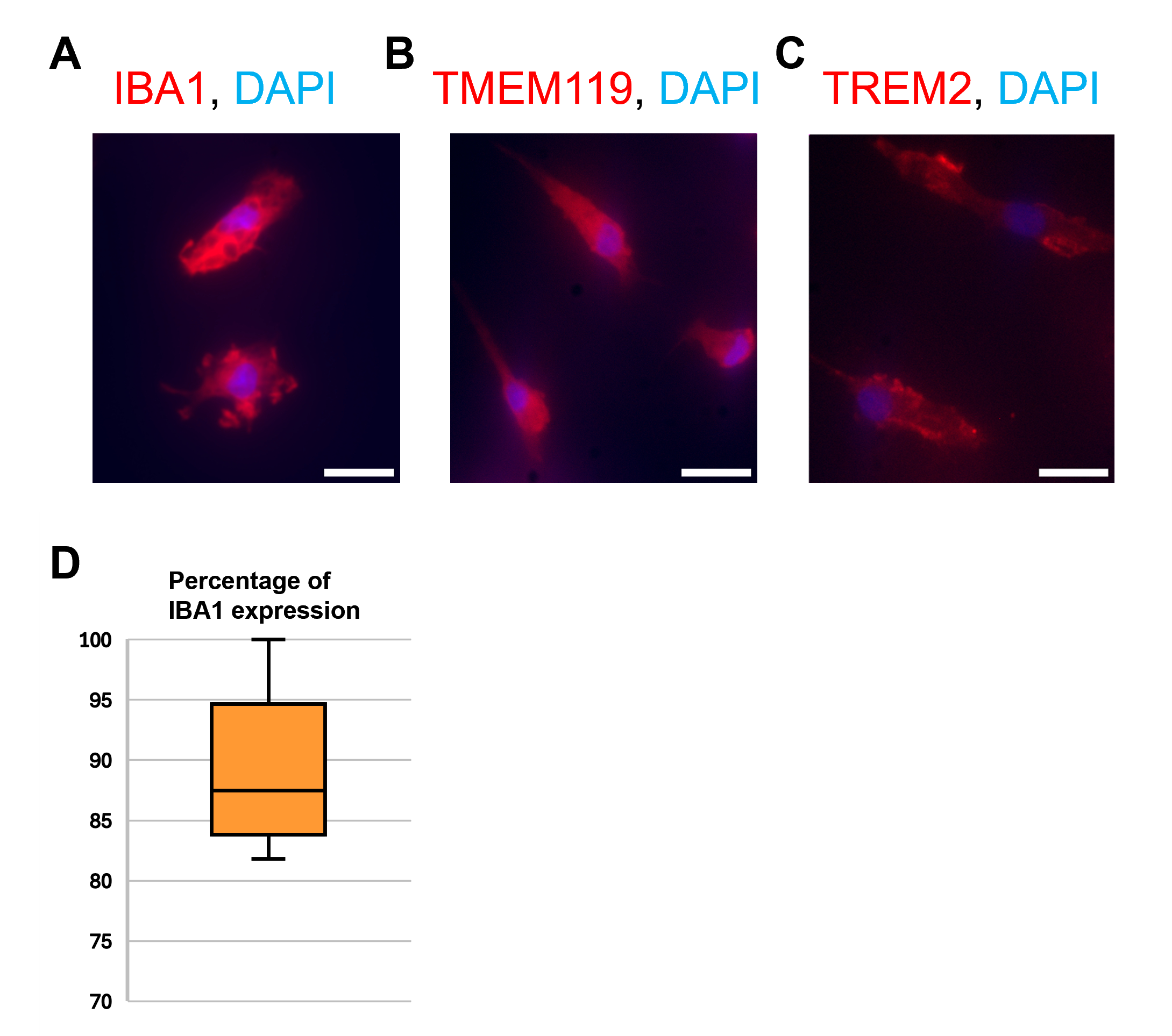

Supplement: Supplementary file 2 — Figure S2: Successful induction of iPSC‐derived microglia (iMG). (A–C) iMGs express common microglia markers IBA1 (red in A), TMEM119 (red in B) and TREM2 (red in C). DAPI marks the nuclei. iMGs generated from iPSCs. Scale bars 20 μm. (D) Percentage expression of IBA1 positive microglia in iMG from the results of Figure 6H,I [file ACEL-24-e70189-s005.tif]

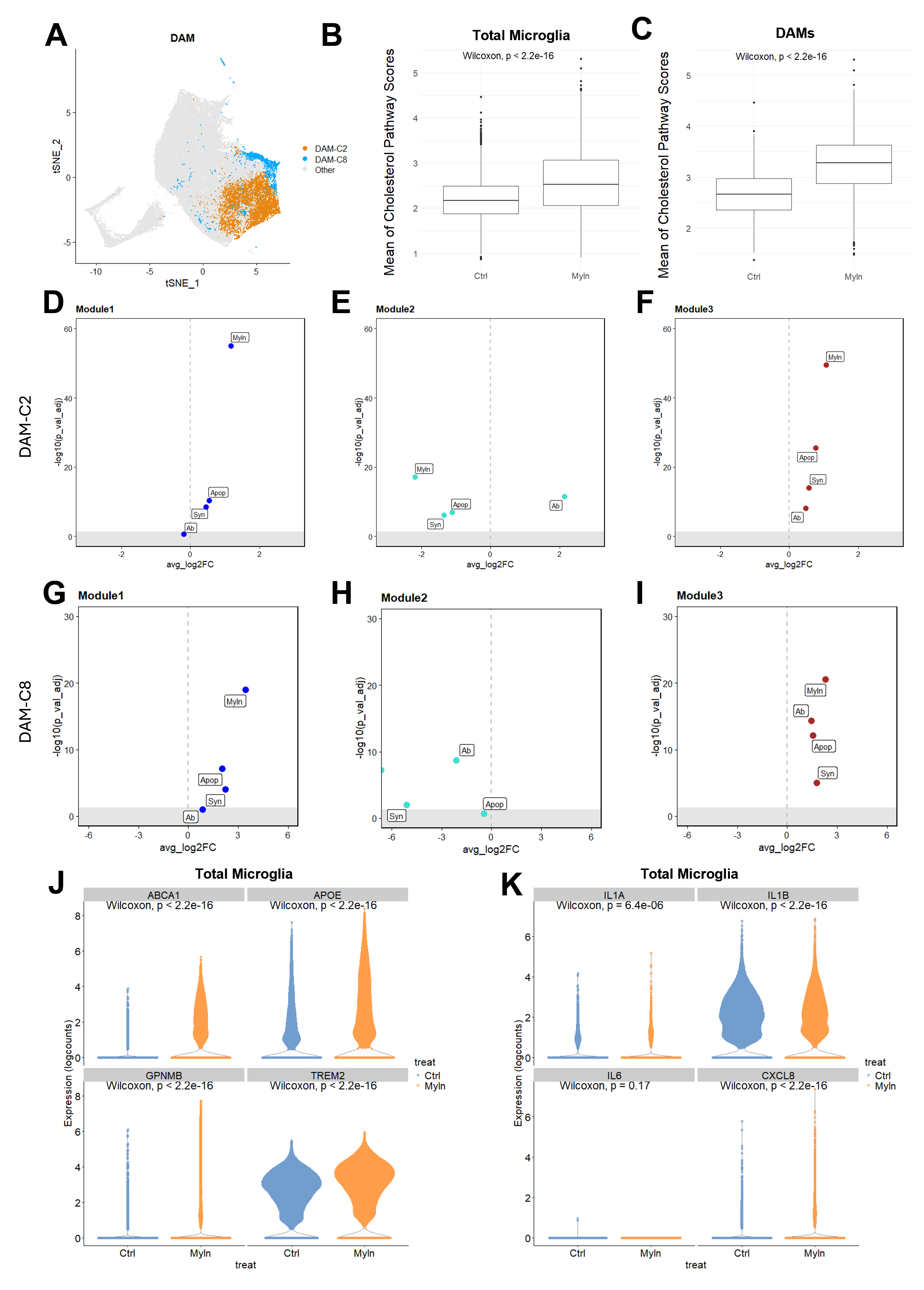

Supplement: Supplementary file 3 — Figure S3: Cholesterol‐senescence signatures increase in microglia treated by myelin. (A) Two DAM clusters are identified after CNS substrate treatment. (B, C) The cell level of cholesterol pathway scores are calculated using the mean of microglia cholesterol‐related (the same set of pathways as Figure 2C) pathways scores together for each individual. Data shown are median ± quartiles and were analyzed using the Wilcoxon signed‐rank test. “Myln” means myelin treatment. “Ctrl” means control. (D–I) The expression levels of the module Eigen genes from Figure 5 in DAM cluster 2 & 8 after CNS substrate treatment. (J) Expression levels of DAM signature genes in the total microglia. (K) Expression levels of inflammatory signature genes in the total microglia. [file ACEL-24-e70189-s008.tif]

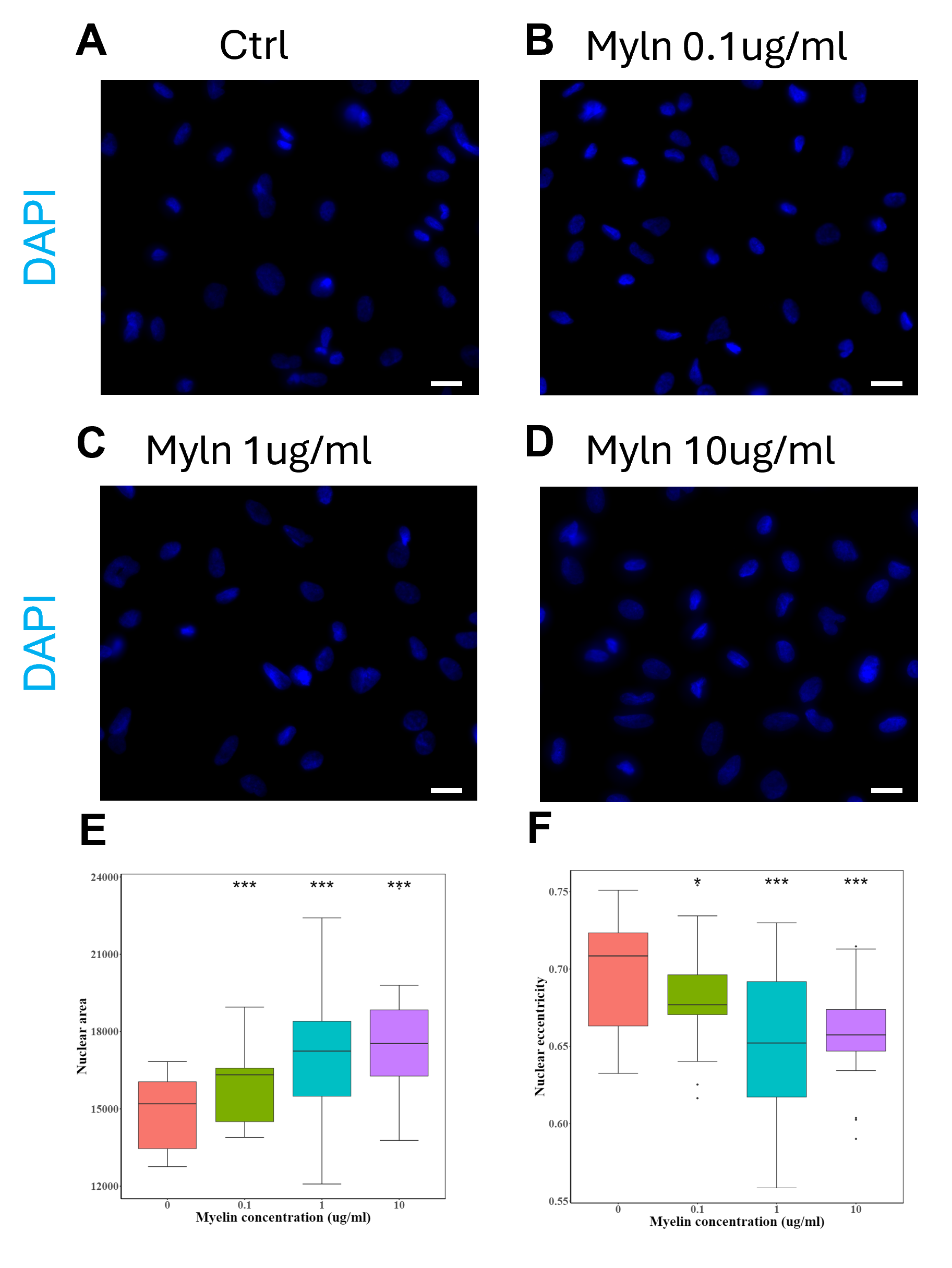

Supplement: Supplementary file 4 — Figure S4: Myelin treated iMGs show changed nuclear morphology. (A–D) Illustration of the nuclear morphology changes of senescent cells. (E, F) myelin treated iMG show decreased eccentricity and increased size. Linear mixed effect model: Variable ~ Treatment+1|Biological replicate, was used to properly account for the structure of the data (n = 18 ROIs: each treatment has 3 biological replicates and 6 technical replicates). Significance is defined by p value (*p < 0.05, **p < 0.01, ***p < 0.001). Box plots in inset show lower and upper hinges at the 25th and 75th percentiles, with whiskers extending to, at most, 1 times the interquartile range (IQR). [file ACEL-24-e70189-s004.tif]

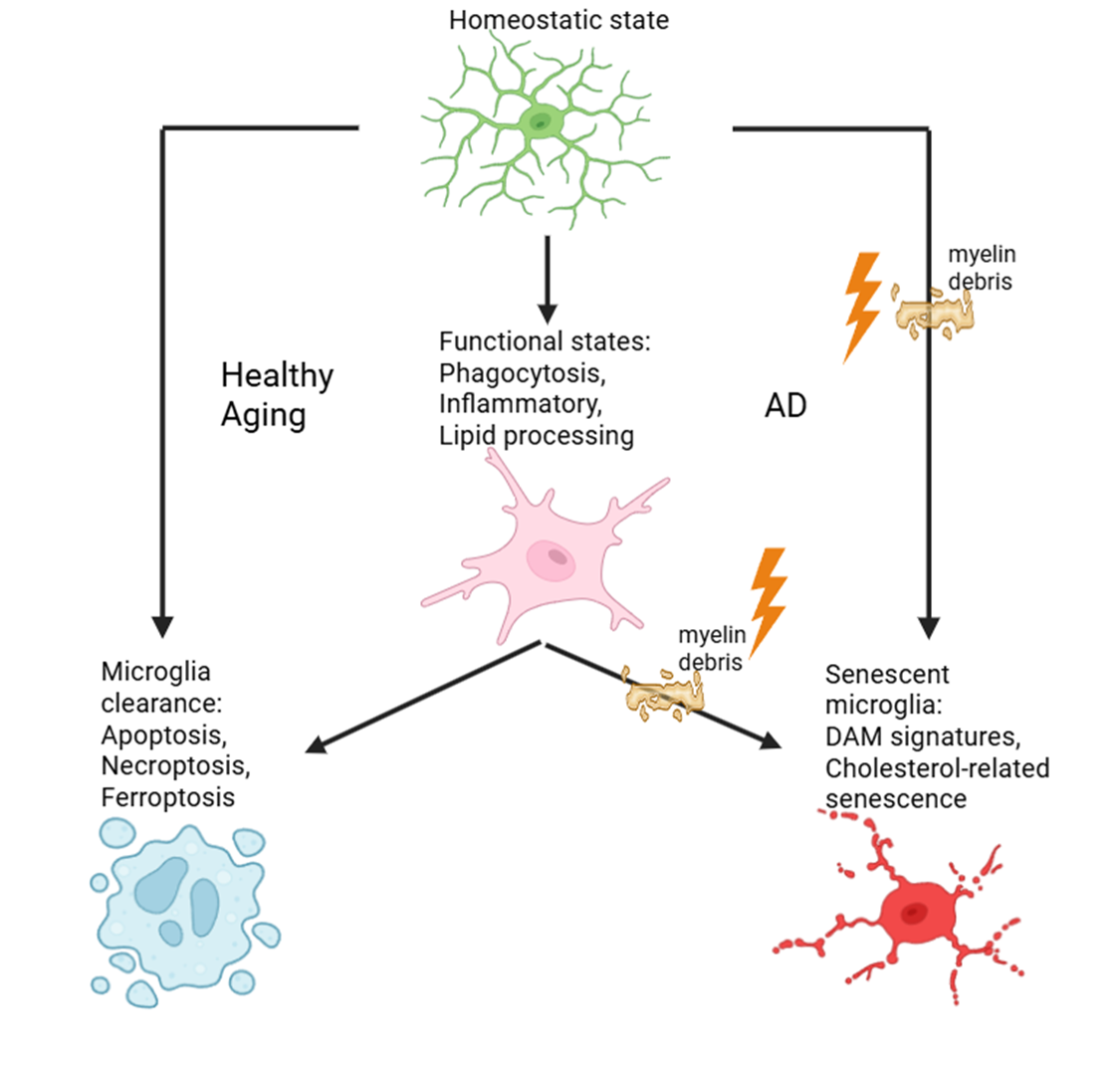

Supplement: Supplementary file 5 — Figure S5: Illustration of the mechanism that myelin debris from oligodendrocytes causes cholesterol dysregulation in microglia, leading to senescent cell states in AD. [file ACEL-24-e70189-s001.tif]

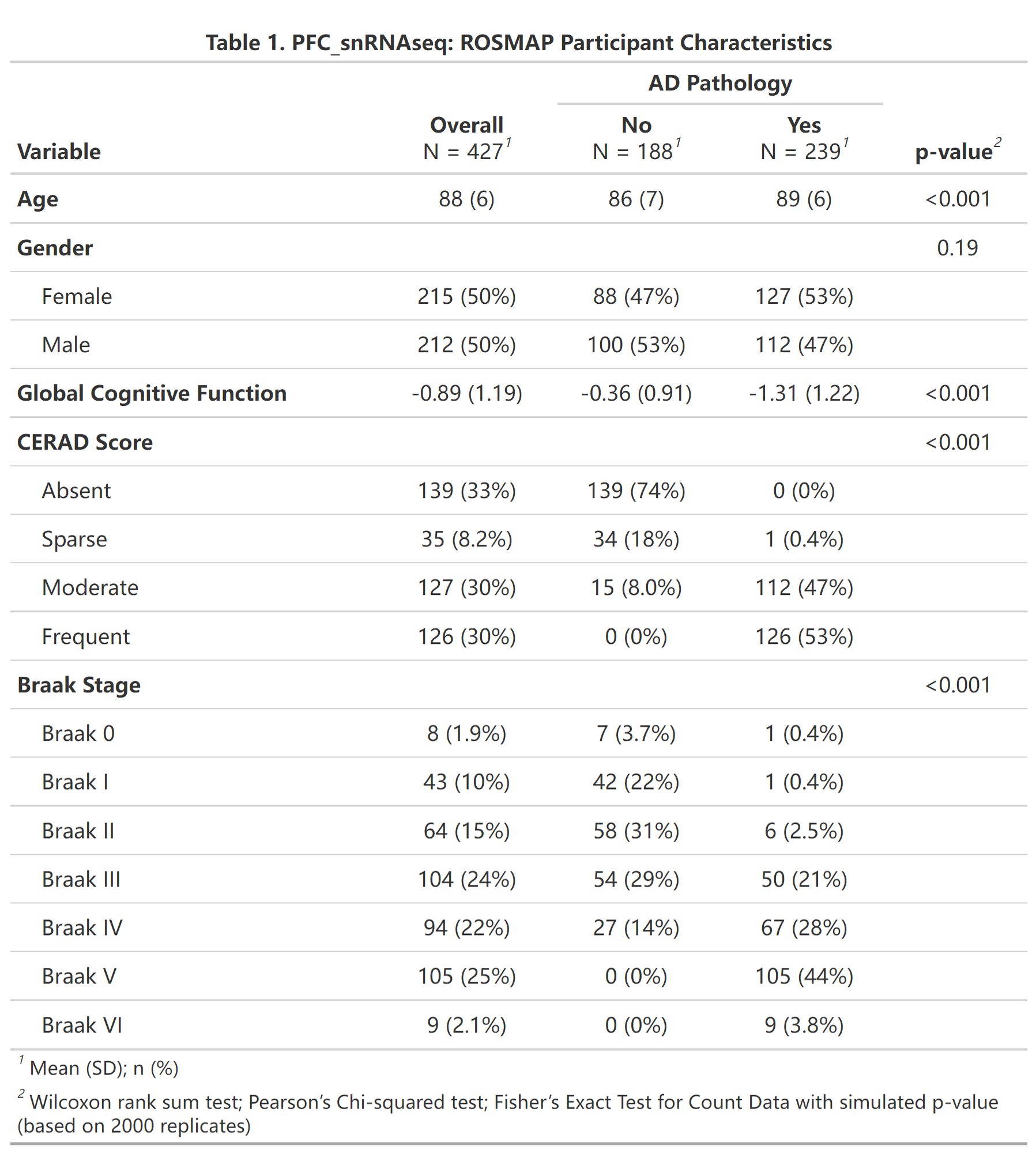

Supplement: Supplementary file 6 — Table S1: ROSMAP participant characteristics for PFC snRNA‐seq data in Figures 1 and 2. [file ACEL-24-e70189-s006.png]

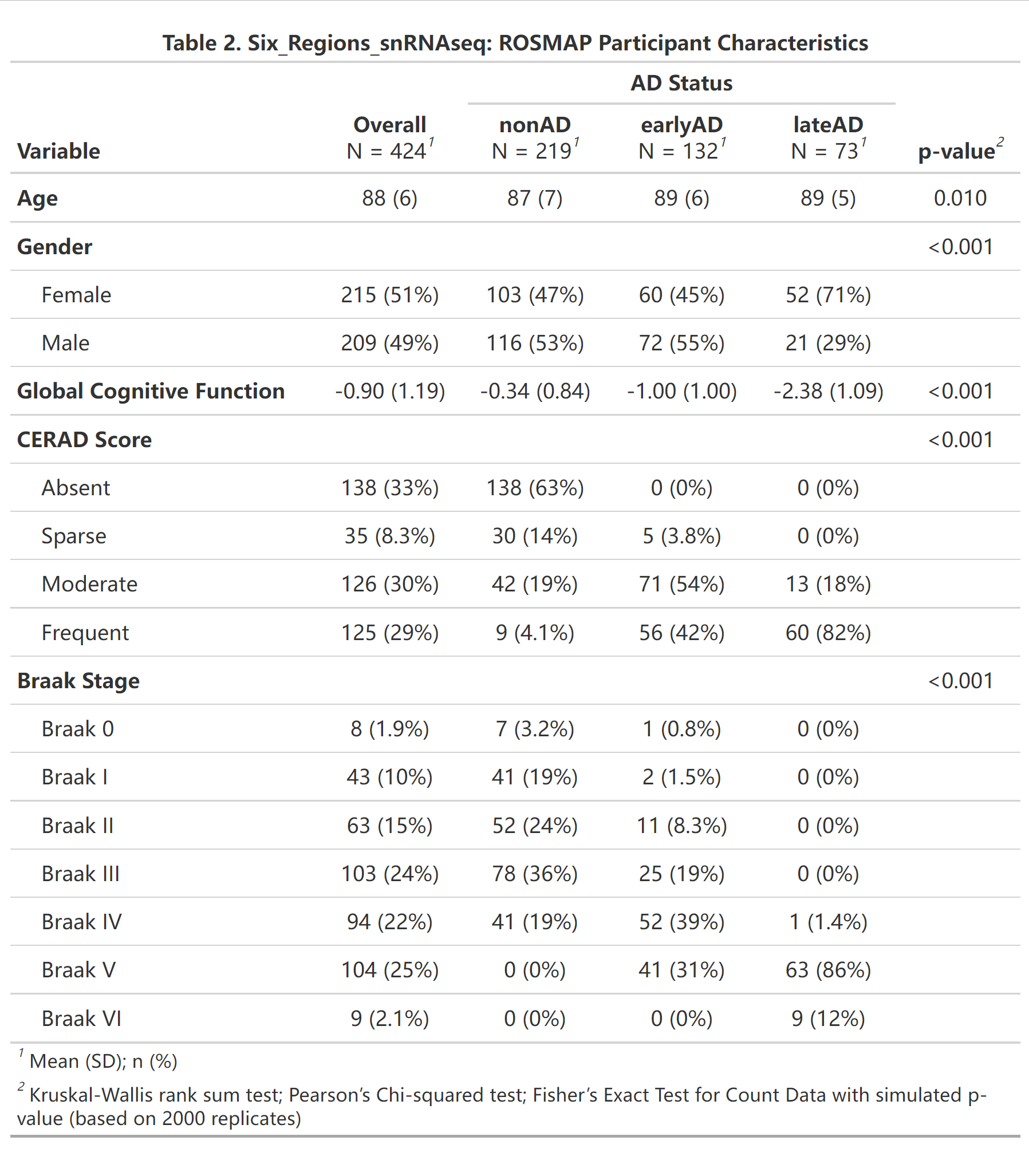

Supplement: Supplementary file 7 — Table S2: ROSMAP participant characteristics for six‐region snRNA‐seq data in Figure 3. [file ACEL-24-e70189-s002.png]

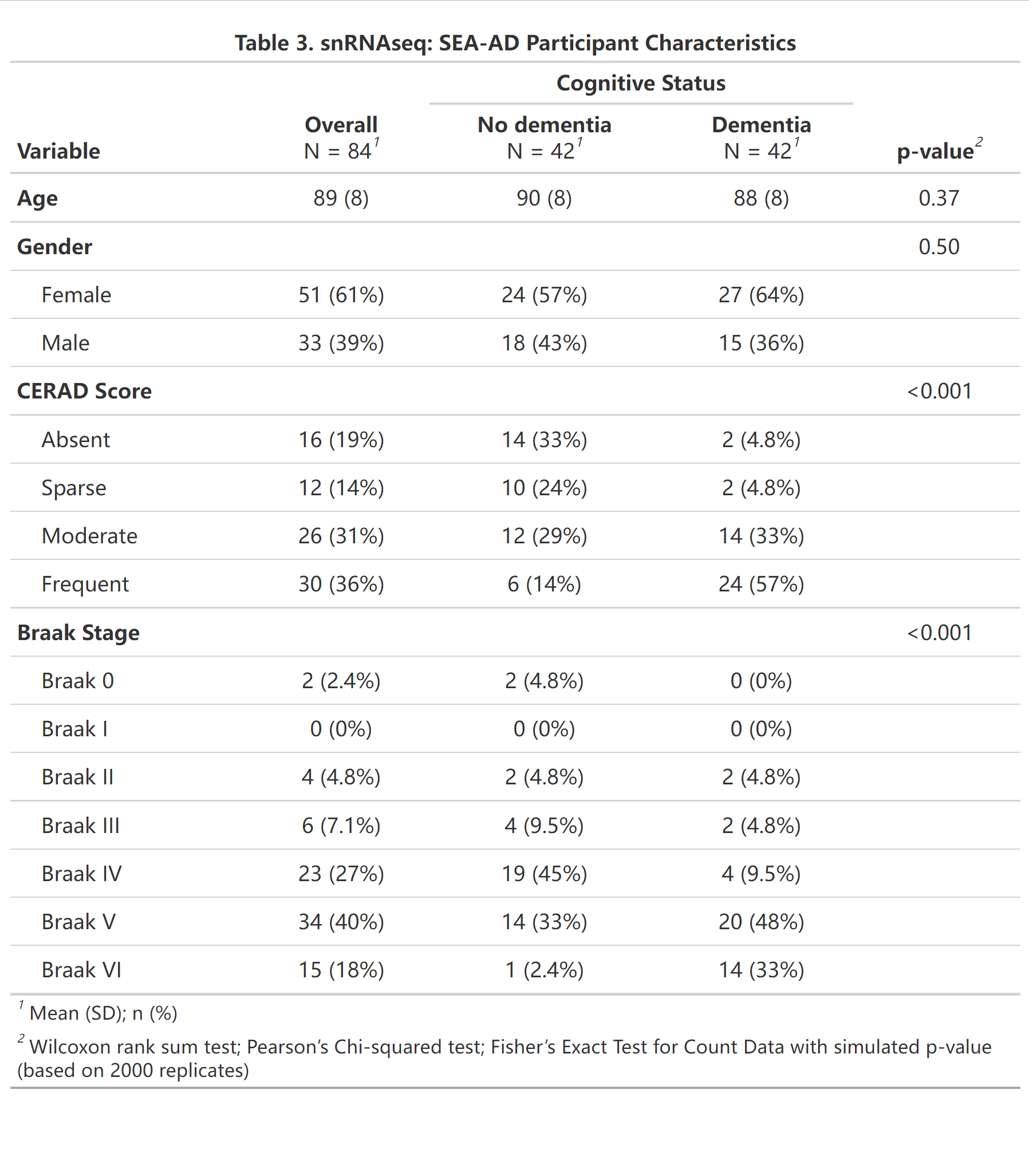

Supplement: Supplementary file 8 — Table S3: SEA‐AD participant characteristics for snRNA‐seq data in Figure 4. [file ACEL-24-e70189-s003.png]
